# An overview of tenofovir and renal disease for the HIV-treating clinician

**DOI:** 10.4102/sajhivmed.v19i1.817

**Published:** 2018-07-17

**Authors:** Willem D.F. Venter, June Fabian, Charles Feldman

**Affiliations:** 1 Wits Reproductive Health and HIV Institute, University of the Witwatersrand, South Africa; 2 Wits Donald Gordon Medical Centre, South Africa; 3 Department of Internal Medicine, Faculty of Health Sciences, University of the Witwatersrand, South Africa; 4 Charlotte Maxeke Johannesburg Academic Hospital, South Africa

## Abstract

Tenofovir disoproxil fumarate (TDF, commonly termed ‘tenofovir’) is the antiretroviral most commonly implicated in antiretroviral-induced nephrotoxicity. As patients on successful antiretroviral therapy (ART) age, their risk for developing renal disease may increase in part because of ART itself, but more importantly, because of HIV-associated and non-HIV-associated comorbidity. Therefore, clinicians need an approach to managing renal disease in people on TDF. TDF as a cause of acute kidney injury (AKI) or chronic kidney disease (CKD) is uncommon, and clinicians should actively exclude other causes (Box 1). In TDF-associated AKI, TDF should be interrupted in all cases, and replaced, or ART interrupted altogether. Tenofovir disoproxil fumarate toxicity can present as AKI or CKD, and as a full or partial Fanconi’s syndrome. TDF has a small but definite negative impact on kidney function (up to a 10% decrease in glomerular filtration rate [GFR]). This occurs because of altered tubular function in those exposed to TDF for treatment and as pre-exposure prophylaxis. Renal function should be assessed using creatinine-based estimated GFR at the time of initiation of TDF, if ART is changed, at 1–3 months, and then ideally every 6–12 months if stable. Specific tests of tubular function are not routinely recommended; in the case of clinical concern, a spot protein or albumin: creatinine ratio is preferable, but in resource-limited settings, urine dipstick can be used. More frequent monitoring may be required in those with established CKD (estimated glomerular filtration rate [eGFR] < 50 mL/min/1.73 m^2^) or risk factors for kidney disease. The most common risk factors are comorbid hypertension, diabetes, HIV-associated kidney disease, hepatitis B or C co-infection, and TDF in combination with a ritonavir-boosted protease inhibitor. Management of these comorbid conditions must be prioritised in this group. If baseline screening eGFR is < 50 mL/min/1.73 m^2^, abacavir (the preferred option), and dose-adjusted TDF (useful if concomitant hepatitis B), zidovudine or stavudine (d4T) remain alternatives to full-dose TDF. If there is a rapid decline in kidney function (eGFR drops by more than 25% and decreases to < 50 mL/min/1.73 m^2^ from of baseline function), or there is new onset or worsening of proteinuria or albuminuria, clinicians should review ART and other potentially nephrotoxic medications and comorbidity and conduct further testing if indicated. If kidney function does not improve after addressing reversible causes of renal failure, then referral to a nephrologist is appropriate. In the case of severe CKD, timeous referral for planning for renal replacement therapy is recommended. Tenofovir alafenamide, a prodrug of tenofovir, appears to have less renal toxicity and is likely to replace TDF in future.

## Introduction

Kidney disease in clinical medicine is a challenge and is often diagnosed only when it becomes very severe. Presentation can be insidious and very non-specific, with multiple possible and overlapping aetiologies. Histology, often needed for definitive diagnosis, requires expertise both for biopsy and for the pathology interpretation. Treatment requires some estimation of the likely aetiology, and access to renal replacement therapy (dialysis and transplant) is often not available in much of the world affected by HIV.

Tenofovir disoproxil fumarate (‘tenofovir’; TDF) is the major antiretroviral implicated in renal disease in first-line antiretroviral therapy (ART). Although other antiretrovirals, especially the protease inhibitors, have been implicated in various forms of kidney disease, and may even potentiate the toxicity of TDF, the fact that tens of millions of people in both resource-rich and resource-limited countries are on TDF translates into large numbers of patients potentially at risk of kidney disease.^1^

The first instance of nephrotoxicity ascribed to TDF was described in the year of the drug’s registration in the United States, in 2001^1^; this was a case with acute kidney injury (AKI), nephrogenic diabetes insipidus and Fanconi’s syndrome. Since then, many studies have described the nephrotoxic potential of the drug. However, separating the impact of TDF on kidney function in clinical practice, especially in the context of ill patients, where multiple concurrent potentially nephrotoxic insults may be seen, is complex.

## Kidney disease and HIV in resource-limited settings

As with much disease epidemiology in resource-limited countries, there are limited data on the background prevalence of chronic kidney disease (CKD) disease in the general population. A recent systematic review called attention to the very poor quality of data on the prevalence of CKD in sub-Saharan Africa.^2^ There has been recent attention paid to non-communicable diseases in resource-limited countries, although it is unclear what proportion of these are attributable to CKD, where it is often compartmentalised with ‘other non-communicable diseases’.^3^ However, the impact on population health is likely to be substantial as CKD is an independent risk factor for cardiovascular disease (CVD) and also considered as a CVD ‘accelerator’.^4^ The recent Global Burden of Disease Study identified kidney disease as a major and increasingly important factor in global health.^5^ Diseases that impact on the prevalence of kidney disease, such as obesity and type 2 diabetes mellitus, appear to be on the rise in some studies, and, as life expectancy increases, it is likely that the prevalence of CKD will, therefore, grow.^6^ With specific relevance to sub-Saharan Africa, Black patients in many studies appear to have a greater burden of CKD, including those with HIV infection.^7^ Late presentation remains a challenge in the region, because of the non-specific symptoms, the absence of screening and prevention programmes for detecting early stages of kidney disease and the limited access to pathology laboratories for testing.^8^

HIV is independently associated with several manifestations of renal disease, including HIV-associated nephropathy (HIVAN).^8,9^ HIV-associated nephropathy manifests as rapidly progressive renal disease, associated with heavy proteinuria, and appears to be because of direct HIV infection of renal tissue. The diagnosis is definitively made by renal biopsy, with characteristic features involving the full kidney architecture. HIV-associated nephropathy occurs relatively early on in the course of HIV disease, with studies showing average CD4+ counts over 200 cells/µL at biopsy diagnosis. In the United States, it disproportionately affects Black people.^10,11^ HIV-associated nephropathy is strongly associated with homozygosity for the APOL1 gene variant associated with more rapid progression of renal disease to end-stage renal failure. This was originally identified in African-Americans and has been demonstrated in South Africans with HIV infection. Other forms of HIV-related renal disease include various immune complex-mediated nephropathies, glomerulonephropathies and renal disease associated with thrombotic thrombocytopenic purpura (TTP).^8^

HIV-related complications, and the drugs used to treat them, account for a substantial proportion of renal manifestations seen in clinical care. Clinical presentations may be acute or chronic, or an acute on chronic episode of kidney injury. HIV-related infections and neoplasms may be implicated in AKI through hypoperfusion from sepsis and dehydration, direct infection or infiltration of renal parenchyma, and urinary outflow obstruction from collections, calculi, tumours or lymphadenopathy. Medications used to treat HIV and related infections have extensive renal toxicity, including antibiotics (commonly, antituberculous drugs, amphotericin B, aminoglycosides and gancyclovir, although almost all classes of antibiotics can be associated with renal effects) and ART (most commonly TDF, as well as atazanavir, indinavir and ritonavir, implicated in renal stone development and interstitial nephritis).^12^

## Literature review of tenofovir disoproxil fumarate toxicity

Initial cell culture, animal and clinical registration studies of TDF suggested that the drug had a good safety profile with regard to renal function, with minimal influence on renal tubule mitochondrial function.^13,14^ However, extensive clinical use and post-marketing surveillance suggested that renal effects occur and may be serious. The difference in results between many of the clinical trials and subsequent observational studies almost certainly reflects the fact that unselected patients, probably those who were sicker and exposed to other renal insults, were excluded from clinical trials.^15^ There is a definite impact of TDF on tubular function, although this is usually not clinically apparent, unless exacerbated by risk factors discussed below. In addition, there is a predictable but small decrease in GFR associated with long-term TDF use. A 2010 meta-analysis involving 17 large studies and over 10 000 patients found that TDF was associated with a statistically significant decrease of creatinine clearance of 3.92 mL/min and a risk of AKI, but no evidence of tubular dysfunction, with wide heterogeneity across studies.^16,17^

### Tenofovir disoproxil fumarate handling in the kidney

Excretion of TDF is almost entirely via the kidney, through glomerular filtration and an active tubule excretion mechanism, and it is excreted into the urine unchanged. There is no interaction with the CYP450 pathway.

**FIGURE 1:**
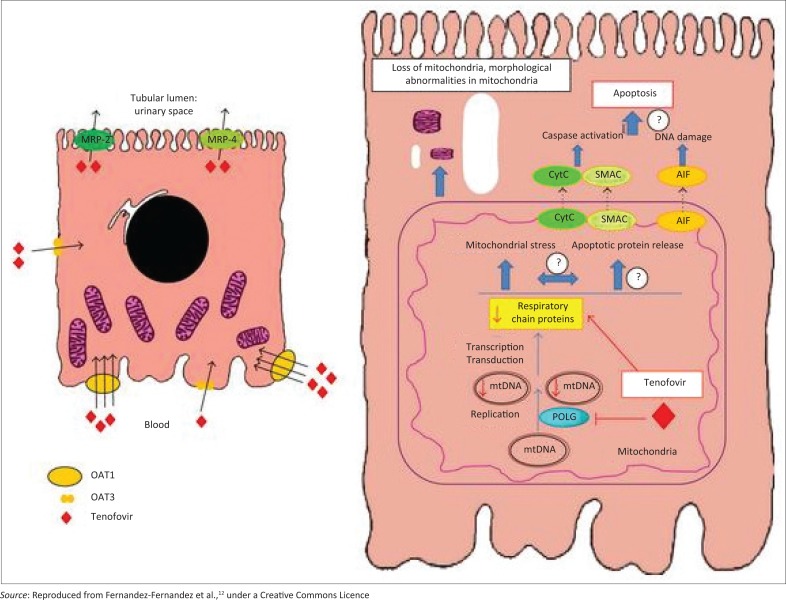
Tubular handling of tenofovir.

Tubular secretion is estimated at 20% – 30% of total excretion of TDF and involves initial organic anion transporter-mediated (OAT-mediated) entry into the proximal tubule, using prominently OAT1 and assisted OAT3 (Figure 1). These OAT transmembrane mediators, only initially described in the late 1990s, have increasingly been found to be important for a variety of therapeutic drug, hormone and endogenous compound modifications, with broad specificity.^14,18^ Extrusion is then mediated by multidrug resistance proteins (MRP), MRP-4 and MRP-2, into the tubular lumen, and a polymorphism in genes coding for MRP has been implicated in tenofovir toxicity.^19^ TDF has a similar structure to adefovir and cidofovir (the three comprise a class of drugs called acyclic nucleotides), both of which carry substantial nephrotoxicity, accumulate in the kidney and have complex interactions with these transporters.^12^ All three are both substrates and inducers of MRP. A decreased glomerular filtration rate (GFR) has been shown to be associated with increased intracellular concentrations of TDF, through increased OAT1 activity. Medications, including probenicid, didanosine, ritonavir and non-steroidal anti-inflammatory drugs (NSAIDs), and synthetic nucleoside analogues herpetic antivirals such as acyclovir that alter OAT and MRP effects may influence intracellular concentration and toxicity of these compounds.^12,20^

The actual mechanism of TDF-induced intracellular toxicity is thought to be via mitochondrial depletion and structural change, including size and shape changes, and leakage of mitochondrial proteins into the cytosol, with resultant DNA damage, which may even induce apoptosis of the cell. These are the changes that are seen in the more studied cidofovir in proximal tubular cells.

The proximal tubule is responsible for the reabsorption of glucose, uric acid, amino acids, small proteins (such as vitamin D-binding protein and β2-microglobulin) and phosphate, the secretion of hydrogen ions and the synthesis of calcitriol. Damage to the proximal tubule leads to wasting of these elements in the urine, renal tubular acidosis and vitamin D deficiency, all of which may be components of the tubular dysfunction described with partial or complete Fanconi’s syndrome. The impact on vitamin D and phosphate is thought to account for the bone manifestations seen with TDF.^21^

### Clinical studies of tenofovir disoproxil fumarate renal toxicity: Decreases in estimated glomerular filtration rate and tubular damage

Animal studies demonstrated tubular toxicity, manifested as electrolyte, protein and glucose loss, as well as rises in urea and creatinine, at very high doses. However, previous human studies of TDF did not show any signs of clinical Fanconi’s syndrome, possibly because of the many exclusion criteria characterising pharmaceutical registration studies; early toxicity case studies tended to involve patients with low muscle mass, patients with pre-existing renal dysfunction and those who were heavily pretreated with antiretrovirals, with resolution of toxicity on withdrawal of TDF.^14^ Subsequently, many cases of tubular dysfunction have been described in case and observational studies, as well as in post-marketing surveillance, including from the manufacturers’ own expanded access programme, which found elevations in creatinine in 2.2% of patients, and severe renal events in 0.5% of patients.^14,16,17,21,22,23^ In addition, TDF has been associated with cases of AKI, usually with evidence of proximal tubular damage, and may occasionally require renal replacement therapy. Recovery may be incomplete in as many as half of patients.^24^ Histology in these cases includes chronic tubular-interstitial scarring, with tubular atrophy and interstitial fibrosis.^24^ TDF levels, both the peak concentration and area under the curve (AUC), are significantly increased with renal dysfunction, leading to the recommendation that all patients receive an assessment of kidney function, with dose adjustment or switching to an alternative agent, if estimated glomerular filtration rate (eGFR) is < 50 mL/min/1.73 m^2^.^14^ Studies have suggested that lower CD4+ cell counts, elevated baseline creatinine levels, older age, concomitant nephrotoxins and comorbidities, the use of didanosine and boosted protease inhibitors, and lower body mass are risk factors for renal toxicity.^14^

Renal disease is staged according to the KDIGO (Kidney Disease Improving Global Outcomes) criteria using eGFR as follows: stage 1 disease: > 90 mL/min/1.73 m^2^ with other evidence of kidney disease (e.g. a single kidney or albuminuria); stage 2 disease: between 60 mL/min/1.73 m^2^ and 89 mL/min/1.73 m^2^; stage 3 disease: between 30 mL/min/1.73 m^2^ and 59 mL/min/1.73 m^2^; stage 4 disease: between 15 mL/min/1.73 m^2^ and 29 mL/min/1.73 m^2^; and stage 5 disease: < 15 mL/min/1.73 m^2^. Chronic kidney disease is defined as an eGFR < 60 mL/min/1.73 m^2^, which includes stages 3, 4 and 5, because the hazard of cardiovascular events and all-cause mortality increase exponentially below 60 mL/min/1.73 m^2^.^25^ There appears to be a consistent slight drop (up to 10%) in eGFR with the use of TDF, across multiple studies,^26,27,28,29,30,31,32,33,34^ and appears to be more marked during the first years of exposure, and more marked at higher eGFRs. One study suggested that this decrease was largely irreversible,^30^ but the difference was of doubtful clinical significance. Studies have been conducted looking at patients who have initiated TDF in the presence of renal dysfunction and have shown that while the majority of patients did not deteriorate during study follow-up, pre-existing stage 2 (eGFR between 60 mL/min/1.73 m^2^ and 89 mL/min/1.73 m^2^) and stage 3 (30–59 mL/min/1.73 m^2^) renal disease were associated with progression to renal failure on TDF,^35,36^ although one study actually demonstrated improvement on TDF in patients with severe (< 30 mL/min/1.73 m^2^) renal failure. TDF use was not associated with renal failure in a small study of hospitalised patients in Zambia.^37^

Oral pre-exposure prophylaxis (PrEP) is used to prevent HIV infection in high-risk groups and currently involves either TDF alone or, more commonly, with emtricitabine. A large number of clinical and observational trials, involving a large number of HIV-negative patients, have demonstrated PrEP renal safety, although again confirming the slight drop in GFR seen in the trials involving HIV-positive patients; however, selection criteria, even in the observational trials, have almost always excluded people with abnormal renal function and/or those who were additionally exposed to nephrotoxins. Finally, these patients were, by virtue of being HIV-negative, often relatively healthy.^38,39,40^ One large study in HIV-negative patients demonstrated a small but significant decrease in eGFR, but this decrease was not progressive, as opposed to the studies in HIV-positive populations.^41^

Overdose with TDF in combination with other antiretrovirals has been described in isolated case reports and most have resolved without any residual kidney abnormalities or with transient rises in creatinine, although one case of permanent kidney damage has been described.^42,43,44^

## Kidney function testing

The assessment of kidney function in clinical practice is complex; symptoms to trigger precise testing are non-specific in most cases and usually occur once significant renal function loss has occurred. Many tests are insensitive to mild dysfunction or may even be normal in the face of significant tubular damage. Finally, many tests are affected by clinical context (such as low muscle mass) or have not been validated in specific populations, particularly in sub-Saharan Africa.

**FIGURE 2:**
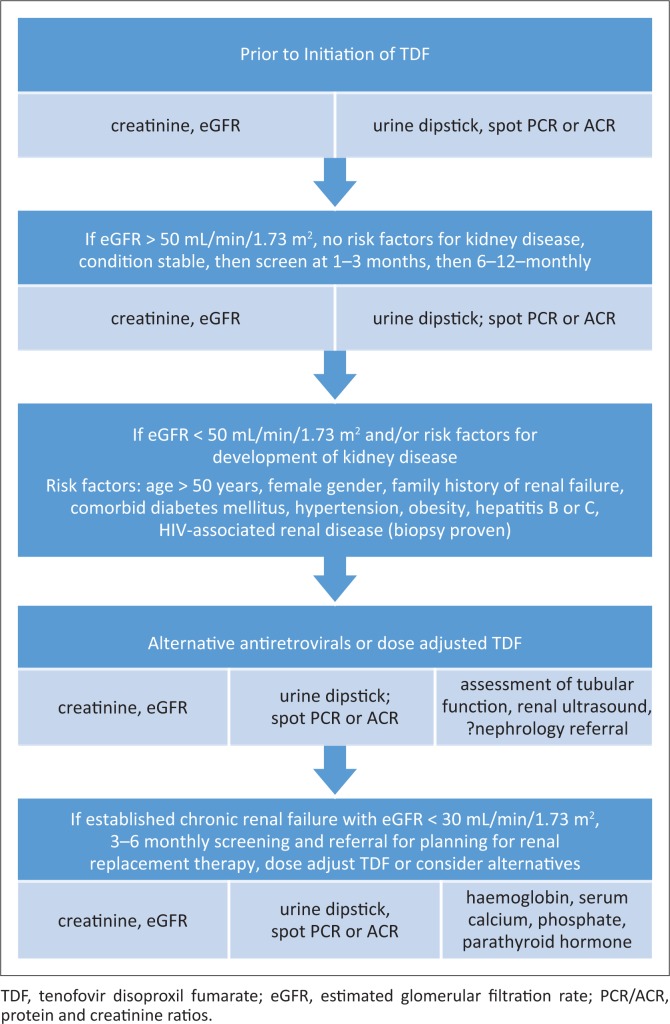
Proposed renal screening for those on tenofovir disoproxil fumarate.

Routine testing of kidney function has significant programmatic implications, especially where laboratory infrastructure is limited. The DART study, in which 75% of the participants received TDF (in combination with zidovudine [ZDV] and lamivudine), suggested that routine monitoring of kidney function was unnecessary, as it did not contribute to clinical outcomes, even though patients had very advanced HIV disease (starting median CD4+ of 86 cell/µL) and low body mass index.^45,46^ However, all guidelines continue to recommend monitoring, although frequency of monitoring varies. One potential solution may be to stratify those receiving TDF according to the presence or absence of kidney disease and their risk for CKD, and then use these criteria to inform kidney function testing (Figure 2).

### Glomerular filtration rate

Glomerular filtration rate is considered the best measure of kidney function. Gold standard measurements for GFR include the clearance of inulin, (51) Cr-EDTA and iohexol, known as measured GFR (mGFR), but these are cumbersome, impractical and expensive – thus very rarely used in clinical practice. Cystatin-C is emerging as an alternative marker and is available in selected South African laboratories, but has yet to enjoy widespread use, partly as it has not been validated locally. As an alternative, certain endogenous markers have been identified, such as creatinine, which is cheap and relatively easy to measure. Creatinine-based estimating equations have been derived from various studies that predict mGFR, such as Cockroft and Gault (C+G), CKD-EPI Collaboration and Modification of Diet in Renal Disease (MDRD). Because creatinine can vary based upon age, gender, ethnicity and dietary protein intake, these equations adjust for the effect of these variables. However, none of the estimating equations for GFR have been developed for populations in sub-Saharan Africa. This is problematic, as studies have proven that these equations perform poorly in the region and are in fact worsened by using the adjustment for ‘race’ that is applied for African-Americans.^47^

Creatinine is almost exclusively derived from muscle breakdown (a small amount is derived from ingested meat). The fact that it is processed at a relatively constant rate, filtered freely by the glomerulus, with little tubular secretion and no absorption, makes it a useful marker of nephron overall function.

There is a debate regarding which calculation to use, especially as their accuracy has not been established in HIV-positive or sub-Saharan African populations. Common eGFR equations including the MDRD or C+G formulae have been criticised for underestimating renal impairment in people with low muscle mass, an independent risk factor for TDF nephrotoxicity. The MDRD was developed in those with renal failure and correlates fairly well when eGFR is < 60 mL/min/1.73 m^2^, but is less accurate if renal function is more preserved. A recent review has been even more broadly critical, saying that extrapolating GFR from plasma creatinine is ‘an inaccurate method’.^48^ The conclusions are supported by moderately strong scientific evidence. However, these equations are widely used as initial screening tests, because they are cheap, and alternatives are either expensive or complex, and almost never validated in local populations.^48^ More recently, the CKD-EPI equation has been shown to perform as well as the MDRD equation when eGFR is < 60 mL/min/1.73 m^2^ and is more accurate than MDRD when eGFR is > 60 mL/min/1.73 m^2^. The equation uses creatinine and adjusts for age, gender and race and has become the preferred equation for studies in which the participants have mostly normal kidney function. This was demonstrated in a cohort of Spanish HIV-positive patients^49^ and is now used as the preferred calculation by the influential European AIDS Clinical Society (EACS) Guidelines.^50^ The KDIGO group recently suggested that the serum creatinine-based CKD-EPI should be the measurement used.^51^

### Tubular

Renal tubular dysfunction may be associated with a preserved GFR, although it seems likely that eventually renal function will be affected. The various components of Fanconi’s syndrome are as follows:

- hypophosphataemia – this alone (‘isolated hypophosphataemia’) or with the other components
- glycosuria, with normal serum glucose
- renal proteinuria, especially loss of β2-microglobulin
- hypouricaemia
- aminoaciduria
- decreased bone mass or osteomalacia.

HIV registration studies have used proxy markers for tubular dysfunction, including urine dipstick screening for haematuria, proteinuria, glycosuria and leucocyturia; spot protein or albumin:creatinine ratios; plasma retinol-binding protein; B2-microglobulin:creatinine ratios; and fractional excretion of uric acid and phosphate. Urinary β2-microblobulin is inversely associated with body weight in patients on TDF and improves on withdrawal of the drug, suggesting that it is a useful marker of renal tubular dysfunction.^52^

Tests which may be useful for assessing the effects of long-term renal tubular dysfunction include plasma phosphate, urine for glucose, dual-energy X-ray absorptiometry (DEXA) and clinical outcomes such as chronic renal disease and osteoporotic fractures, although guidance for the rational use of the laboratory tests is lacking.

Spot urine protein and creatinine ratios (PCR/ACR) are useful, and simple to perform, and correlate well with 24 hour protein collections, which in turn correlate with renal disease progression. However, protein may be from the glomerulus or tubules, so is not specific for tubular toxicity. Conventional dipstick testing measures albumin, not other proteins, so may miss tubular dysfunction. It is important for clinicians to understand the significance of albuminuria in the setting of chronic renal disease. Albuminuria, in its own right, is an independent predictor for cardiovascular and all-cause mortality (irrespective of GFR), which is why it is now included in the definition of CKD. This makes spot ACR measurement compelling as a general screen, even when not associated with tubular dysfunction.

## Conclusion

TDF has, over the last 15 years, become a critical component of HIV treatment (Table 1). TDF is a safe drug in terms of renal clinical safety, at least in the short to medium term, although it is associated with a small but predictable decrease in eGFR. Bone clinical effects of tubular dysfunction are still being documented, although osteomalacia was significant in one study.^21^ As patients age on successful ART and start to acquire multiple comorbidities, and as age itself is a risk factor for TDF toxicity, more patients acquiring renal disease while on TDF is inevitable. The consistent observation that pre-existing renal disease seems to be a major predictor of TDF toxicity may suggest that baseline testing may allow some mitigation of the small risk of renal harm; some studies question whether creatinine monitoring after a normal baseline test is necessary, in the absence of other factors to suggest renal disease. It may be prudent, in resource-limited situations, to limit this to those with comorbidities. The phasing out of didanosine and the limited use of boosted protease inhibitors, both implicated in TDF nephrotoxicity, may mean that previous toxicity seen with this combination in resource-limited countries will be rare in our region.

**TABLE 1:** Tenofovir disproxil fumarate usage timeline in South Africa.

Year	Tenofovir disproxil fumarate usage
2001	Licenced by US FDA; talks on patent transfer informally occurred in South Africa between Gilead Sciences and local South African activists
2002	Licenced in Europe
2003	Gilead acquires Triangle, holder of emtricitabine (FTC) licence
2004	Truvada, a combination of TDF and FTC, launched
2005	Aspen, a South African generic manufacturer, signs licensing and distribution agreement with Gilead; Gilead signs further agreements from 2006 onwards
2006	Submission of registration dossier to SA regulatory body, MCC; WHO recommends ‘moving away’ from stavudine; MSD launches Atripla, a single tablet containing TDF, FTC and efavirenz; TDF features in popular South African soap opera as alternative to stavudine
2007	MCC licences TDF after activist group TAC advocate for registration; rapidly becomes private sector preferred first-line drug; WHO adds TDF to Essential Medicines List; Gilead signs licencing deal with technology transfer to 11 generic manufacturers
2009	Gilead joins Unitaid Medicines Patent Pool
2010	TDF becomes part of first-line treatment in South African state programme; delayed MCC registration of new suppliers of active pharmaceutical ingredients for TDF cited as major reason for continued high prices
2011–2012	Country-wide stock outs because of supply line failures
End 2011	Aspen registers Tribuss, a generic version of Atripla; multiple other generics follow
2012	New South African antiretroviral tender worth almost $1 billion allows for broad access to fixed dose combinations containing TDF; almost 2 million South Africans on ART, supply line failures of fixed dose combination drugs decrease; Truvada licenced for PrEP by FDA
2015	PrEP (using TDF/FTC) registered by MCC in South Africa
2017	TAF registered in the United States; studies commence in South Africa on TAF use in first-line therapy; almost 4 million South Africans on TDF

*Source*: Aspen;^55^ Business Day;^56^ Ford et al.^57^

FDA, Food and Drug Administration; TDF, tenofovir disoproxil fumarate; MCC, Medicines Control Committee; TAC, Treatment Action Campaign; PrEP, pre-exposure prophylaxis; TAF, tenofovir alafenamide fumarate.

If creatinine is measured and eGFR calculated to monitor toxicity, most guidelines recommend repeating measurements in the first 1–3 months of treatment and then 6-monthly monitoring (Southern African HIV Clinicians Society, British HIV Association, European AIDS Clinical Society). Some guidelines advocate urine dipstick testing, with more rigorous tubular function testing if positive for glucose or protein. Measuring ACR should be considered if comorbidities are present, and risk factors managed aggressively.

Withdrawal of TDF is prudent in the context of both AKI and CKD, as it is almost impossible to rule out at least some contribution to renal disease. Recovery occurs over weeks to months and is often incomplete. It is probable that the earlier TDF damage is detected and the drug stopped, the recovery will be more complete. Alternatives to full dose TDF, especially in the context of renal failure, include dose reducing TDF itself; abacavir, which requires no dose adjustment; ZDV, which only requires dose adjustment at extremely low eGFRs; or stavudine (d4T), which requires dose adjustment. However, both ZDV and d4T are associated with substantial toxicity, as well as twice-daily dosing, in the medium to long term. For chronic renal failure, the same drug substitutions hold. A further practical option is to dose reduce TDF with alternative day dosing (creatinine clearance 30 mL/min–49 mL/min) or twice weekly dosing (creatinine clearance 10 mL/min–29 mL/min).^53^

Interrupting TDF in hepatitis B chronically infected patients is a difficult decision, especially when alternative drugs are not available (dose-adjusted entecavir is an option, if available, or lamivudine with pegylated interferon alpha). Interruptions may be associated with hepatitis because of increased replication of the hepatitis B virus, which may cause significant morbidity and even mortality, especially in those with cirrhosis. In an acute situation, clinicians may have to weigh this risk against further renal damage before interrupting TDF.

Tenofovir alafenamide fumarate (TAF), another prodrug of the active form of TDF, has a far lower plasma exposure and has demonstrated less impact on the laboratory markers of tubular function, eGFR and bone mass. The drug is currently in various clinical trials and has been licenced in several resource-rich countries. Drug interactions with rifampicin remain a concern, but TAF is likely to replace the current form of TDF in the next few years.^54^

Box 1 Rapid clinical approach for an HIV patient with kidney dysfunction taking antiretrovirals.

- **Clinical history considerations:** Ingestion of any herbal toxin, over the counter medication (in particular, non-steroidal anti-inflammatories), concomitant potential nephrotoxins (especially antibiotics, including rifampicin); current ART regimen and date of introduction; previous baseline eGFR; history of kidney disease (AKI and/or CKD) or conditions associated with increased risk for CKD (diabetes mellitus, hypertension, etc.); and recent acute illness (gastroenteritis, pneumonia, TB and urinary tract infection).
- **Clinical assessment:** Sepsis, dehydration and evidence of outflow obstruction (lymph nodes, masses and oedema).
- **Tests:**
  ■ **Kidney function:** Creatinine; eGFR; serum calcium and phosphate; urine dipstick – may see evidence of tubular wasting, with glycosuria (important to establish normal plasma glucose); urine microscopy may demonstrate protein casts; urine cultures for TB and mycobacteria; and spot PCR or ACR.
  ■ **Clinically indicated tests to document concomitant pathology:** Full blood count with smear (sepsis, thrombocytopenic thrombotic purpura, neoplasm and TB leukaemoid reactions); liver function testing; blood cultures; and aspirates of lymph nodes or masses.
  ■ **Abdominal or renal ultrasound:** May demonstrate obstructive lesions (always consider if evidence of immune reconstitution syndrome, IRIS).
  ■ **Renal biopsy:** Only if specifically indicated (requires nephrology opinion).
